# New Developments in the Synthesis of EMICORON

**DOI:** 10.3390/ht7030022

**Published:** 2018-08-29

**Authors:** Massimo Pitorri, Marco Franceschin, Ilaria Serafini, Alessandro Ciccòla, Claudio Frezza, Armandodoriano Bianco

**Affiliations:** 1 Dipartimento di Chimica: Università di Roma “La Sapienza”, Piazzale Aldo Moro 5, 00185 Rome, Italy; pitorri.1174810@studenti.uniroma1.it (M.P.); ilaria.serafini@uniroma1.it (I.S.); alessandro.ciccola@uniroma1.it (A.C.); armandodoriano.bianco@uniroma1.it (A.B.); 2 Dipartimento di Biologia Ambientale: Università di Roma “La Sapienza”, Piazzale Aldo Moro 5, 00185 Rome, Italy

**Keywords:** EMICORON, polycyclic aromatic compounds, organic synthesis, synthesis improvements, anticancer activity

## Abstract

This paper reports on the modification of two synthetic steps in the usual protocol used for obtaining EMICORON. EMICORON is a benzo[*ghi*]perylen-diimide, which was synthesized for the first time in our laboratory in 2012, and has shown to have in vivo antitumor activities that interferes with the tumor growth and development using a multi-target mechanism of action. The provided modifications, which involved the reaction times, the reaction conditions, and the work-up procedures, allowed the global yield of the process to be increased from 28% to about 40%. Thus, this new procedure may be more suitable for recovering higher amounts of EMICORON to be used in further preclinical studies.

## 1. Introduction

Polycyclic aromatic compounds with hydrophilic polar side chains are well known to inhibit telomerase [1] and therefore possess anticancer properties [2,3]. In particular, perylene and coronene [4,5] derivatives with further modifications have shown to be highly selective, with good activity.

**Figure 1:**
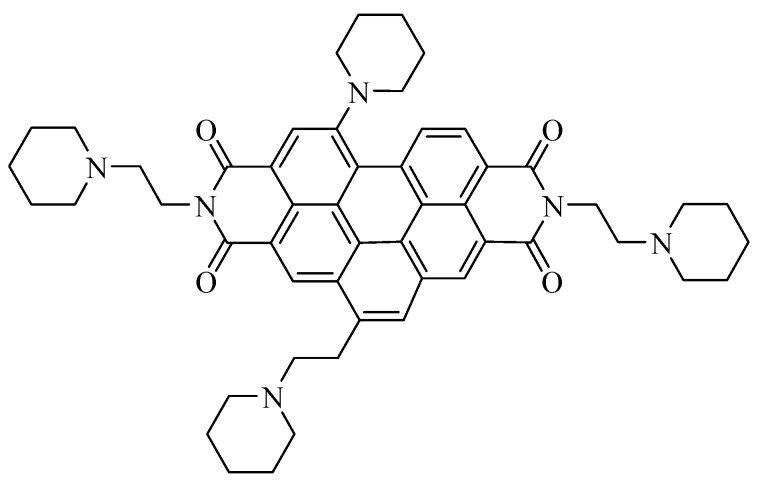
Structure of EMICORON.

EMICORON represents another development of the above-mentioned compounds. It is a polyaromatic compound constituted by a benzo[*ghi*]perylenic core that presents two ethyl-piperidine chains on the major axis, together with one ethyl-piperidine chain and one piperidine chain on the minor axis (Figure 1) [6].

**Figure 2:**
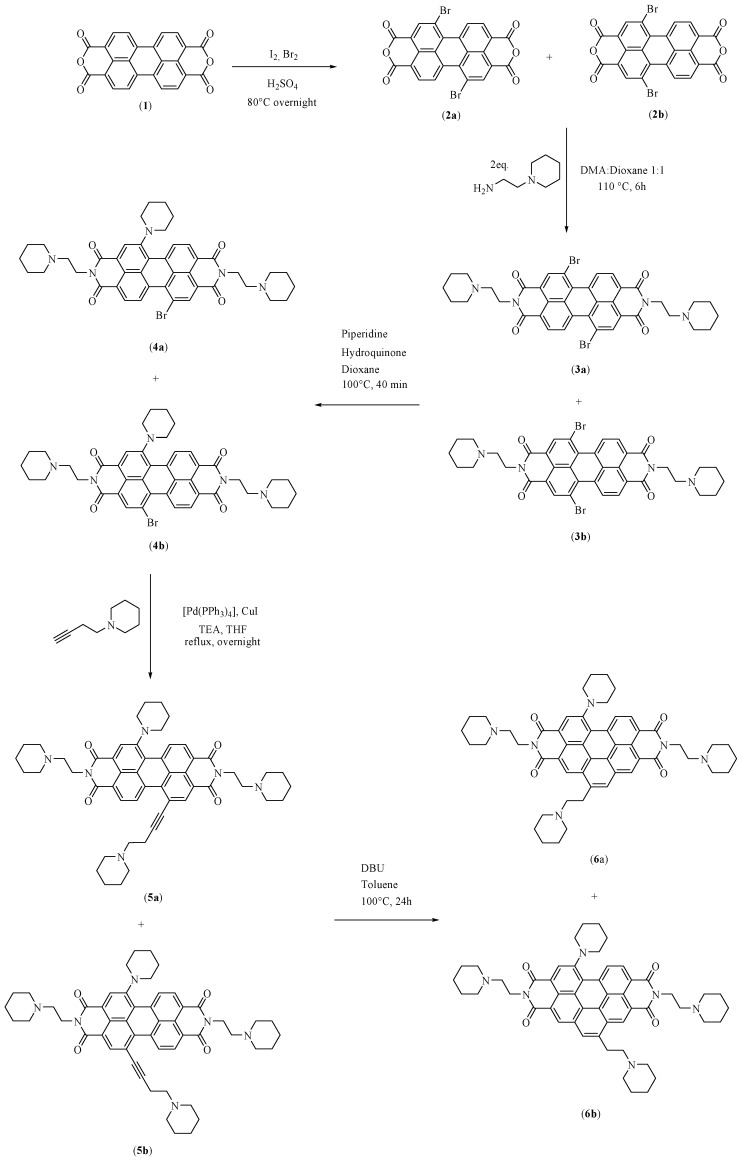
Schematic original protocol for the synthesis of EMICORON.

This compound was synthesized for the first time in our laboratory in 2012 [6]. This first synthesis of EMICORON was carried out through a linear synthesis that comprised five steps [6,7] (Figure 2).

The first step is the most important and involves the bromination of the perylene-3,4:9,10-tetracarboxylic dianhydride to produce two regioisomers that are dibromoderivates of the initial anhydride [7]. The regioselective synthesis of the desiderated regioisomer—1,7-dibromo-3,4:9,10-tetracarboxylic dianhydride (Figure 2, compound **2a**)—has been performed by researchers [8] but without gaining any real advantage. In fact, in terms of yields and atomic efficiency, it is still much more convenient to perform the bromination by following the method reported by Franceschin and co-workers [7], which involves the separation of the obtained regioisomers by classic column chromatography (CC) only after the last step of the synthesis protocol. In spite of our various attempts, the yield of the synthesis obtained by this procedure has always been found to be equal to around 28%.

EMICORON has shown to exhibit in vivo antitumor activity, with the compound being able to reduce the growth of colon-rectal cancer in rats [9]. This result was demonstrated to be achieved using a multi-target mechanism of action. At high doses, EMICRON acts as a telomerase inhibitor [6]; at minor concentrations, it induces apoptosis in tumor cells by rapidly triggering extensive damage to telomeric DNA because of the displacement of the telomeric protein protection of telomeres 1 known as POT1 [6]. 

EMICORON has also been shown to bind G-rich oncogene promoters and downregulate the expression of genes [10]. Moreover, in both in vitro and in vivo studies, it has been observed that EMICRON acts with other conventional antitumor drugs in a synergic effect [11].

Because of all these synthetic and pharmacological reasons, the main aim of this work was to improve the total yield of the synthesis in order to get more amounts of EMICORON for further preclinical studies.

## 2. Materials and Methods

### 2.1. Materials

All the reagents, anhydrous solvents, and deuterated solvents were purchased from Sigma-Aldrich (Saint Louis, MO, USA) and used with no further purification.

Silica gel for classical column chromatography (40–63 μm particle size) was purchased from Fluka Analytical (St. Gallen, Switzerland).

### 2.2. Instrumentation

^1^H-NMR spectra were recorded on a Varian (now Agilent Technologies) (Santa Clara, CA, USA) Mercury 300 MHz spectrometer. 

CDCl_3_ (deuteron-chloroform) was used as deuterated solvent, and the chemical shifts were expressed from TMS (s, 0.00 ppm).

### 2.3. Synthesis

#### 2.3.1. Synthesis of *N*,*N*′-bis[2-(1-piperidino)-ethyl]-1,7-dibromoperylene-3,4:9,10-tetracarboxylic diimide (PIPER-Br)

Under argon atmosphere, 5.001 g of a mixture of the two isomers of dibromoperylene-3,4:9,10-tetracarboxylic dianhydride in ratio 8:1 (*w*/*w*) were dissolved at room temperature in 18 mL of anhydrous DMA and 18 mL of anhydrous dioxane. At this point, 2.6 mL of 1-(2-amminoetyl)-piperidine were added. The reaction mixture was stirred at 110 °C for 12 h. After cooling, cold water (4 °C) was added. Then, 6.522 g of the final product—a mixture of the two isomers in ratio 8:1 (*w*/*w*)—were obtained and isolated by filtration as a red solid. The yield was 93%.

#### 2.3.2. Synthesis of *N*,*N*′-bis[2-(1-piperidino)-ethyl]-1-(1-piperidinyl)-7-bromoperylene-3,4:9,10-tetracarboxylic diimide (PIP-PIPER-Br)

Under argon atmosphere, 6.011 g of the products obtained from the previous reaction were dissolved in 30 mL of piperidine and stirred at 80 °C for 30 min. Cold water at 4 °C was added, and the crude product was extracted with dichloromethane. The organic phase was, then, washed with water until the aqueous layer was neutralized. The product of interest—a mixture of the two isomers in ratio 8:1 (*w*/*w*)—with a weight of 4.502 g and a yield of 71% was separated from the crude product by means of column chromatography on silica gel using a mixture of dichloromethane and methanol 95:5 (*v*/*v*) as eluting system.

### 2.4. NMR Data of All the Synthetic Compounds

*N*,*N*′-bis[2-(1-piperidino)-ethyl]-1,7-dibromoperylene-3,4:9,10-tetracarboxylic diimide (Figure 2, compound **3a**): ^1^H-NMR (300 MHz, CDCl_3_) δ: 9.44 (2H, d, *J* = 8.1 Hz, aromatic H), 8.88 (2H, s, aromatic H), 8.66 (2H, d, *J =* 8.1 Hz, aromatic H), 4.36 (4H, t, *J =* 7.0 Hz, N_imidic_-CH_2_), 2.67 (4H, t, *J =* 7.0 Hz, N_piperidine_-CH_2_), 2.57–2.53 (8H, m, CH_2piperidine_), 1.62–1.54 (8H, m, CH_2piperidine_), 1.47–1.41 (4H, m, CH_2piperidine_) (Spectrum reported in Supplementary Material as Figure S1).

*N,N*′-bis[2-(1-piperidino)-ethyl]-1-(1-piperidinyl)-7-bromoperylene-3,4:9,10-tetracarboxylic diimide (PIP-PIPER-Br) (Figure 2, compound **4a**): ^1^H-NMR (300 MHz, CDCl_3_) δ: 9.41–9.34 (2H, m, aromatic H), 8.80 (1H, s, aromatic H), 8.57 (1H, d, *J =* 8.2 Hz, aromatic H), 8.49–8.46 (2H, m, aromatic H), 4.40–4.34 (4H, m, N_imidic_-CH_2_), 3.46–3.34 (2H, m, C_ar_-N_piperidine_-CH_2_), 3.03–2.95 (2H, m, C_ar_-N_piperidine_-CH_2_), 2.71–2.55 (12H, m, N_piperidine_-CH_2_ and CH_2piperidine_), 1.89–1.42 (18H, m, CH_2piperidine_) (Spectrum reported in Supplementary Material as Figure S2).

## 3. Results and Discussions

The improvement in the synthesis of EMICORON was obtained by the modification of two reactions corresponding to the second and the third steps in the original protocol [6,7].

**Figure 3:**
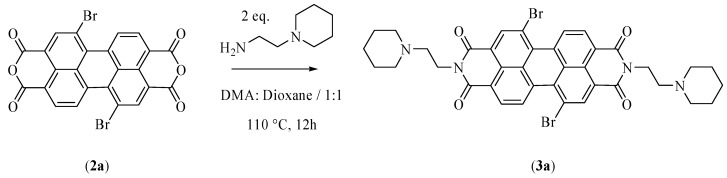
Scheme of the reaction modifications for step two.

The second step leads to the formation of *N*,*N*′-bis[2-(1-piperidino)-ethyl]-1,7-dibromoperylene-3,4:9,10-tetracarboxylic diimide (PIPER-Br) (Figure 2, compound **3a**). In this study, the reaction time was increased from 6 to 12 h with respect to the previous protocol. Moreover, the precipitation of the product was performed by adding cold water at 4 °C to the reaction mixture instead of water at room temperature (Figure 3). This new methodology was able to improve the yield of this reaction step by about 20%.

The third step leads to the formation of PIP-PIPER-Br (*N*,*N*′-bis [2-(1-piperidino)-ethyl]-1-(1-piperidinyl)-7-bromoperylene-3,4:9,10-tetracarboxylic diimide (Figure 2, compound **4a**). The original protocol involved the use of a mixture of dioxane and piperidine in ratio 1:1 (*v*/*v*), together with hydroquinone at 100 °C, for 40 min. The presence of hydroquinone was essential to promote the inhibition of some radical reactions that could happen in similar conditions, leading to dehalogenated collateral products [12,13]. Moreover, it was not possible to isolate the final product of this step as pure compound in such conditions, and a further classical CC on silica gel was necessary in order to also remove some reaction products of the hydroquinone.

**Figure 4:**
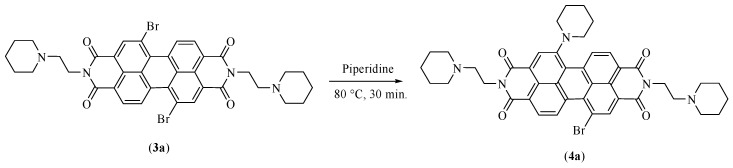
Scheme of the reaction modifications for step three.

By contrast, the new protocol proposed in this study proceeds by totally removing dioxane, and using only piperidine, both as solvent and reagent, and under Argon atmosphere. In such conditions, the reaction was observed to yield in less time and at a lower temperature (Figure 4).

The atomic efficiency of this reaction step was seen to greatly increase with this method. Moreover, the derived products—the desired PIPER-Br (Figure 2, compound **4a**), its regioisomer, and the two corresponding piperidinyl-disubstituted compounds—could be directly used as starting material for the next synthesis step without the need to purify the products by column chromatography. Although the specific yield of this reaction could not be calculated, it increased the global yield for the synthesis of EMICORON, anyway, because the chromatographic passage—which, according to its own nature, is well known to cause the loss of some quantities of the product—was eliminated from the synthetic protocol.

## 4. Conclusions

The modification of the reaction conditions of two previously yield-limiting steps of the synthesis of EMICORON led to an increase in the global yield and avoided the use, in one case, of problematic solvents. Thus, this new protocol may represent a new way of obtaining EMICORON at higher amounts for medicinal purposes.

## Supplementary Materials

The following are available online at http://www.mdpi.com/2571-5135/7/3/22/s1, Figure S1: ^1^H-NMR spectrum of PIPER-Br, Figure S2: ^1^H-NMR spectrum of PIP-PIPER-Br.
