# Supplementary material for: New Developments in the Synthesis of EMICORON
Source: High Throughput. 2018 Aug 29;7(3):22. doi: 10.3390/ht7030022 (PMC6165106; doi:10.3390/ht7030022)
Supplement: Supplementary file 1 [file high-throughput-07-00022-s001.pdf]

Supplementary Materials: **New Developments in the Synthesis of EMICORON**

Massimo Pitorri, Marco Franceschin, Ilaria Serafini, Alessandro Ciccòla, Claudio Frezza and Armandodoriano Bianco

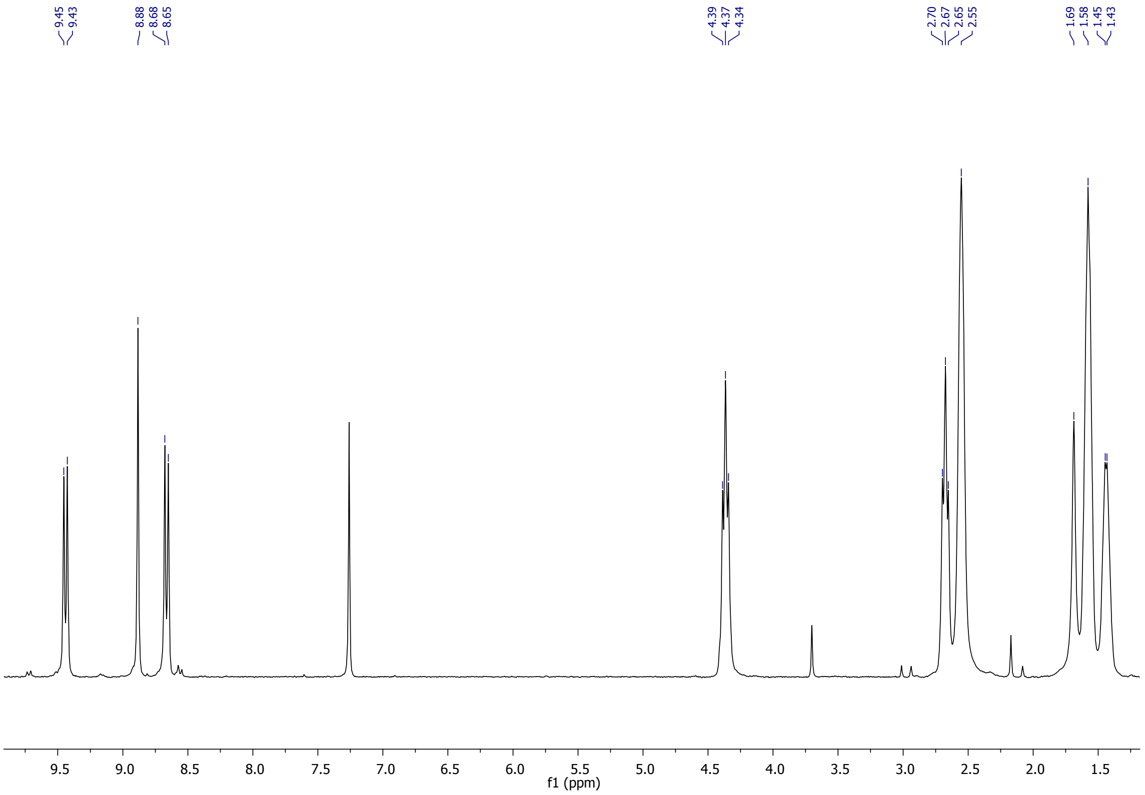

Figure S1. ^1H-NMR spectrum of PIPER-Br.

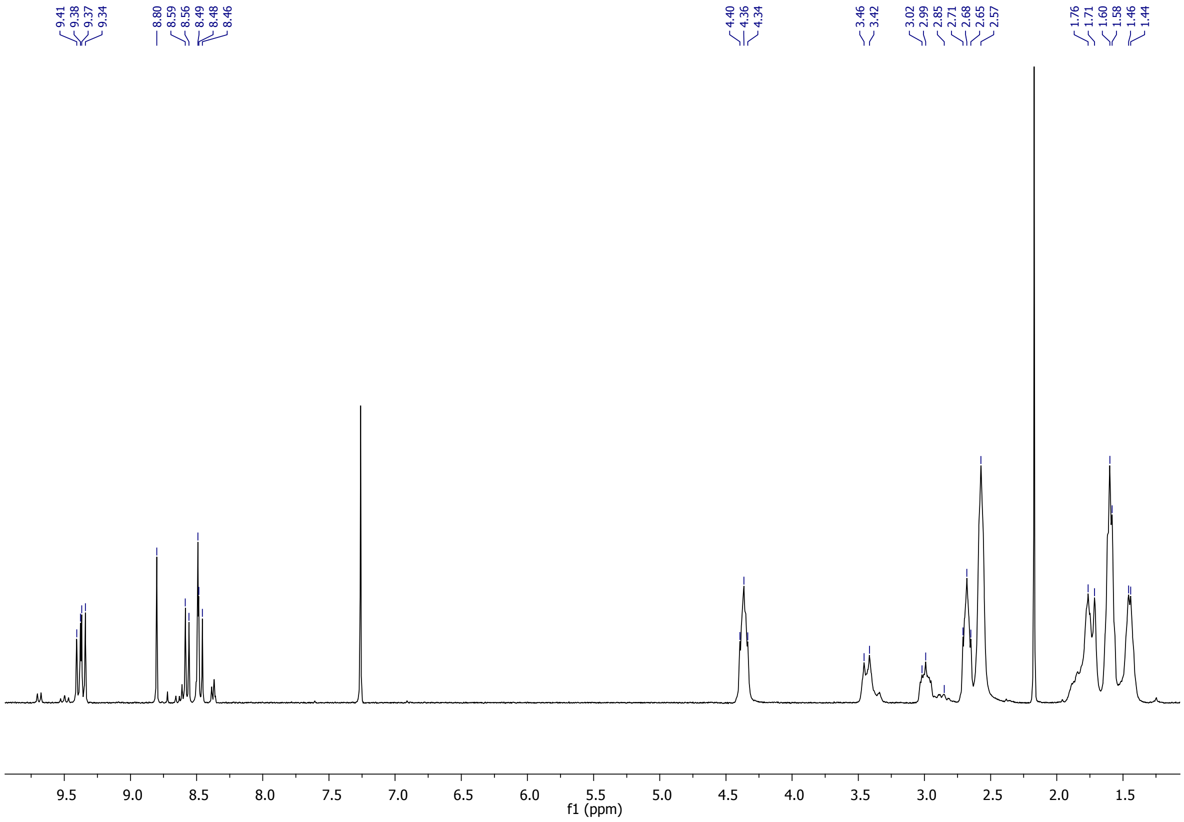

Figure S1. ^{1}H-NMR spectrum of PIP-PIPER-Br.
